# An Evaluation of the Prevalence of Use of High Dose Antipsychotic Therapy Across the General Adult Inpatient Wards and the Psychiatric Intensive Care Unit in Mersey Care NHS Foundation Trust

**DOI:** 10.1192/bjo.2024.479

**Published:** 2024-08-01

**Authors:** Declan Hyland, Roopa Singh, Kerry Dainton

**Affiliations:** Mersey Care NHS Foundation Trust, Liverpool, United Kingdom

## Abstract

**Aims:**

High Dose Antipsychotic Therapy (HDAT) should only be used in exceptional circumstances, as there is little evidence to suggest that higher than recommended doses of antipsychotics are more clinically effective than standard doses, with potential side effects being greater. In practice, there are several clinical scenarios where HDAT may be prescribed and the potential benefits must outweigh the potential risks. NICE guidelines for psychosis and schizophrenia advise that dosages outside the range given in the British National Formulary should be justified and recorded.

This evaluation aimed to determine prevalence of HDAT across the 16 general adult inpatient wards and the Psychiatric Intensive Care Unit (PICU) in Mersey Care NHS Foundation Trust.

**Methods:**

A list of all inpatients admitted to the 16 general adult inpatient wards and to the PICU in the Trust between 17^th^ and 20^th^ of July 2023 was obtained. The electronic prescription record for each patient was scrutinised to determine whether the patient was subject to HDAT and, if so, whether this was due to antipsychotic monotherapy, combination of two or more antipsychotics, or due to regular and as required (PRN) antipsychotic medication.

**Results:**

Of the 215 inpatients on the 16 general adult wards and the PICU, a total of 29 (13.5%) patients were prescribed HDAT. Four wards had no patients on HDAT; one ward had 5 patients on HDAT. Two of the 12 patients on the PICU were on HDAT. Of the 29 HDAT patients, none were on just one regular antipsychotic, 11 were on one regular antipsychotic and one PRN, 11 on two regular antipsychotics only, 4 were on two regular antipsychotics and one PRN antipsychotic, 1 patient was on three regular antipsychotics and 2 patients on three regular antipsychotics and one PRN antipsychotic. Of the 29 HDAT patients, 14 (48%) had schizoaffective disorder, 9 (31%) had schizophrenia, 5 (17%) had bipolar disorder and 1 (4%) had emotionally unstable personality disorder.

**Conclusion:**

Only a minority of inpatients on the general adult wards and the PICU are prescribed HDAT. There was variation in HDAT prescribing across the wards and this may reflect the degree of diagnostic variability of each ward's inpatients. In those patients that are subject to HDAT, there is a need for appropriate baseline physical investigations to be completed and for appropriate monitoring of ECG and relevant blood tests. There is a need to consider whether each HDAT patient has been considered for treatment with clozapine, if appropriate.

